# Does telehealth affect the adherence to ART among patients with HIV? A systematic review and meta-analysis

**DOI:** 10.1186/s12879-023-08119-w

**Published:** 2023-03-17

**Authors:** Elham Davtalab Esmaeili, Hosein Azizi, Saeed Dastgiri, Leila R. Kalankesh

**Affiliations:** 1 Road Traffic Injury Research Center, Tabriz University of Medical Sciences, Tabriz, Iran; 2 ٌWomen’s Reproductive Health Research Center, Tabriz University of Medical Sciences, Tabriz, Iran; 3 Department of Epidemiology and Biostatistics, School of Public Health, Tehran University of Medical Sciences, Tehran, Iran; 4 Tabriz Health Services Management Research Center, Tabriz University of Medical Sciences, Tabriz, Iran

**Keywords:** Digital Health, HIV, Acquired immunodeficiency syndrome, Adherence, Antiretroviral therapy (ART), Telemedicine, Meta-analysis

## Abstract

**Background:**

Several studies have shown different effects of telehealth interventions on adherence to Antiretroviral therapy (ART) among people living with HIV. This study conducted a meta-analysis of Randomized Controlled Trials (RCTs) to estimate the pooled effect of telehealth interventions on the treatment adherence of HIV patients.

**Methods:**

The researchers conducted literature searches in Scopus, PubMed, Web of Science, Google Scholar, and Cochrane Central Register of Controlled Trials databases. In addition, open grey was systematically searched until January 2022 for RCTs around the effects of telehealth on adherence to treatment ART among patients with HIV. Each study’s methodological quality was assessed using the Cochrane Collaboration tool. Pooled Standard Mean Differences (SMD) and Risk Ratio (RR) with 95% CI were calculated using the random effects model.

**Results:**

In total, 12 eligible articles were considered in the present systematic review. A random-effects meta-analysis using 5 RCTs yielded the pooled RR estimate of 1.18 (95% CI: 1.03 to 1.35, p < 0.05); I2 = 0, suggesting the adherence to treatment among patients with HIV who received telehealth intervention was significantly 18% upper than control groups. Moreover, the random effects analysis of SMD showed a positive effect for telehealth with SMR = 0.36 (95% CI: 0.22 to 0.49, p < 0.05); I2 = 91.9%, indicating that telehealth intervention increased ART adherence to the treatment group compared to the control group.

**Conclusion:**

Telehealth intervention as a new modality of health care service delivery could be a valuable strategy to improve ART adherence among patients with HIV. It can strengthen the capacity of HIV care services. On a large scale, telehealth can be utilized as a supplementary component for ART delivery and retention toward successful adherence to the therapy.

## Background

Acquired Immune Deficiency Syndrome (AIDS) is caused by the Human immunodeficiency virus (HIV) [1]. HIV is spreading rapidly worldwide, and it is considered one of the major public health issues. Most people with AIDS are aged 15 and above, leading to social, economic, and political instability [2, 3]. According to the World Health Organization (WHO) report, approximately 38.4 million (33.9–43.8 million) people were living with HIV at the end of 2021. The number of people newly infected with HIV and who died from HIV-related causes worldwide in 2021 was 1.5 million (1.1–2.0 million) and 650 000 (510 000–860,000), respectively. The prevalence of AIDS varies among regions and countries. The WHO African region accounts for most people living with HIV (1 in every 25 adults, and two-thirds of all the people). About 0.7% of 15–49 years old persons have HIV [4].

The Joint United Nations Programme on HIV/AIDS (UNAIDS) was established in 2014 to control the HIV epidemic. According to this program, 90% of HIV patients should be diagnosed, 90% of people diagnosed should be treated with antiviral drugs, and HIV should be suppressed in 90% of the people treated. Proper implementation of this program will lead to the suppression of HIV in 73% of infected people and will be a practical step to ending the epidemic by 2030 [5]. HIV-related mortality in 2020 was 47% fewer than in 2010. This statistic indicates that although the incidence of AIDS has decreased, its transmission continues.

With increasing global access to Antiretroviral therapy (ART), maintaining care and ART remains challenging in controlling the HIV epidemic [6–8]. Adherence to therapy is defined as the degree of compliance of patients’ medication-taking behavior with their health care recommendations, including the number of drugs and the time of taking medications [9]. Various studies reported more than 95% adherence to treatment for optimal viral suppression [7]. Lack of adherence or poor adherence to ART leads to an increase in complications, transfer of infection, health care costs, mortality, and lower quality of life [10, 11]. Poor ART adherence has been attributed to social stigma, forgetfulness, drug side effects, anxiety, depression, substance abuse, and financial problems [12–14]. Advances in information technology, global access to the internet, distance learning, and communication facilities have provided an unprecedented opportunity to scale up the full spectrum of HIV care services through telehealth approaches. Telehealth-based approach to HIV treatment with ART is one of these opportunities.

Although previous studies had suggested using telehealth in the context of HIV to alleviate stigma and increase timely access to care and therapeutic function, the coronavirus disease 2019 (COVID-19) pandemic emerged as a turning point for the utilization of digital technologies such as telehealth in various subjects of medical practice [15–20]. The exponential use of telehealth during this pandemic has been highlighted in several studies [21–24]. Telehealth has also been instrumental during the COVID-19 epidemic in preventing interruption in HIV care, curbing the spread of COVID-19 within the healthcare setting, and closing the existing disparity gap in HIV care [25].

Different studies have been conducted on the effect of telehealth on ART adherence [26–28]. Telehealth can distribute information and services related to HIV care on a large scale via electronic information and telecommunication technologies [29]. It provides long-distance contact between patient and clinician to care, intervene, advise, educate and remind through telecommunication equipment, computer software, mobile apps, social media, websites, chat rooms, games, patient monitoring devices, mobile phones’ short message service, and portable computers [30, 31]. Such an approach is low-cost and also reduces social stigma.

Studies have shown different results of feasibility, acceptability, and effect of telehealth interventions on ART adherence among people living with HIV. Based on the availability of high-quality published, there is a need for precise assessment effects of a telehealth intervention on treatment adherence of HIV patients. This study conducted a meta-analysis of Randomized Controlled Trials (RCTs) to estimate the overall effect of a telehealth intervention on treatment adherence of HIV patients.

## Materials and methods

### Literature search

This study was done following the PRISMA (Preferred Reporting Items for Systematic Reviews and Meta-Analysis) guideline [32]. We designed the research question in the first stage by defining PICO (Population/Problem, Intervention/Exposer, Control, and Outcome). Then we searched studies that examined the effect of a telehealth intervention on treatment adherence of HIV patients. Scopus, PubMed, ISI Web of Science, Google Scholar, Cochrane Central Register of Controlled Trials databases, and open grey were searched until January 2022 for articles published in English without any time limit. Hand searching in the reference list of related articles was conducted additionally. A comprehensive search strategy was developed using the guidance of a librarian. Medical subject heading terms (MeSH), keywords, and text words for each component of the PICO combined using Boolean operators. Finally, “Telemedicine*”, “Mobile Health*”, “Health, Mobile*”, “M Health*”, “Telehealth*”, “E-Health*”, “Therapeutic Adherence and Compliance*”, “Treatment Adherence*”, “Therapeutic Adherence*”,” Acceptability of Health Care*”, “Adherence Medication*”, “Medication Compliance*”, “ART adherence*”, “Antiretroviral adherence*” AND “Human Immunodeficiency Virus*”, “Acquired Immune Deficiency Syndrome Virus*”, “Lymphadenopathy-Associated Virus*”,” HTLV-IV*” were included in the search strategy. In the end, Citations of all fined studies were imported to EndNote 20.1.

### Study selection

After removing the duplicate articles from the search list, the title and abstract of the studies were screened independently by two reviewers (ED and HA). In case of regarding the inclusion of the study, a third reviewer (SD) was consulted.

Studies were selected if they had the following predefined inclusion criteria: (1) all peer-reviewed RCTs with either parallel or crossover designs (2) full text published in the English language; (3) using either viral load or different ways to measure adherence to ART (4) quantitative or mixed-method study design. The studies were excluded if they were (1) observational studies, systematic reviews, and meta-analyses; (2) trials without a control or comparison arm; (3) qualitative study design. Finally, studies that demonstrated self-reported adherence and met other criteria were included in the meta-analysis.

### Data extraction and quality assessment

First author’s family name, publication year, country of origin, setting of trial, study design, method of statistical analysis, number of participants in experiment and control groups, types of interventions, type of control group, duration of follow-up (For articles with more than one time at follow-up, the longest follow-up period was considered) and outcome measuring method were extracted from studies. If a study had more than one arm, each subgroup’s data were considered a separate RCT.

The methodological quality of each study was assessed by the Cochrane Collaboration tool [33]. The main component of this tool included: Random sequence generation, allocation concealment, blinding of participants, personnel and outcome assessment, incomplete outcome data, and selective reporting. Based on this instrument guideline, each study was categorized as ‘good’ if it was low risk in at least three of the items above, ‘weak’ if it was low risk for no or only one item, and ‘fair’ if it was low risk in two items. If a study had more than one arm, each subgroup’s data were considered a separate RCT.

### Statistical analysis

Ten studies met the criteria for inclusion in the meta-analysis. According to the studies on the number of primary studies required for meta-analysis, ten primary studies are enough to perform the analysis [34, 35]. Risk ratio (RR) and 95% confidence intervals and Standard Mean Differences (SMD) and 95% confidence intervals (CI) were expressed as effect sizes. Pooled SMD and OR (Odds Ratio) with 95% CI were calculated using the Der Simonian and Laird method via the random effects model [36]. Cochran’s Q test and I^2^ were performed to assess heterogeneity among the studies [37]. All statistical analyses were performed using STATA software package version 16. (StataCorp, College Station, TX).

## Results

**Fig. 1:**
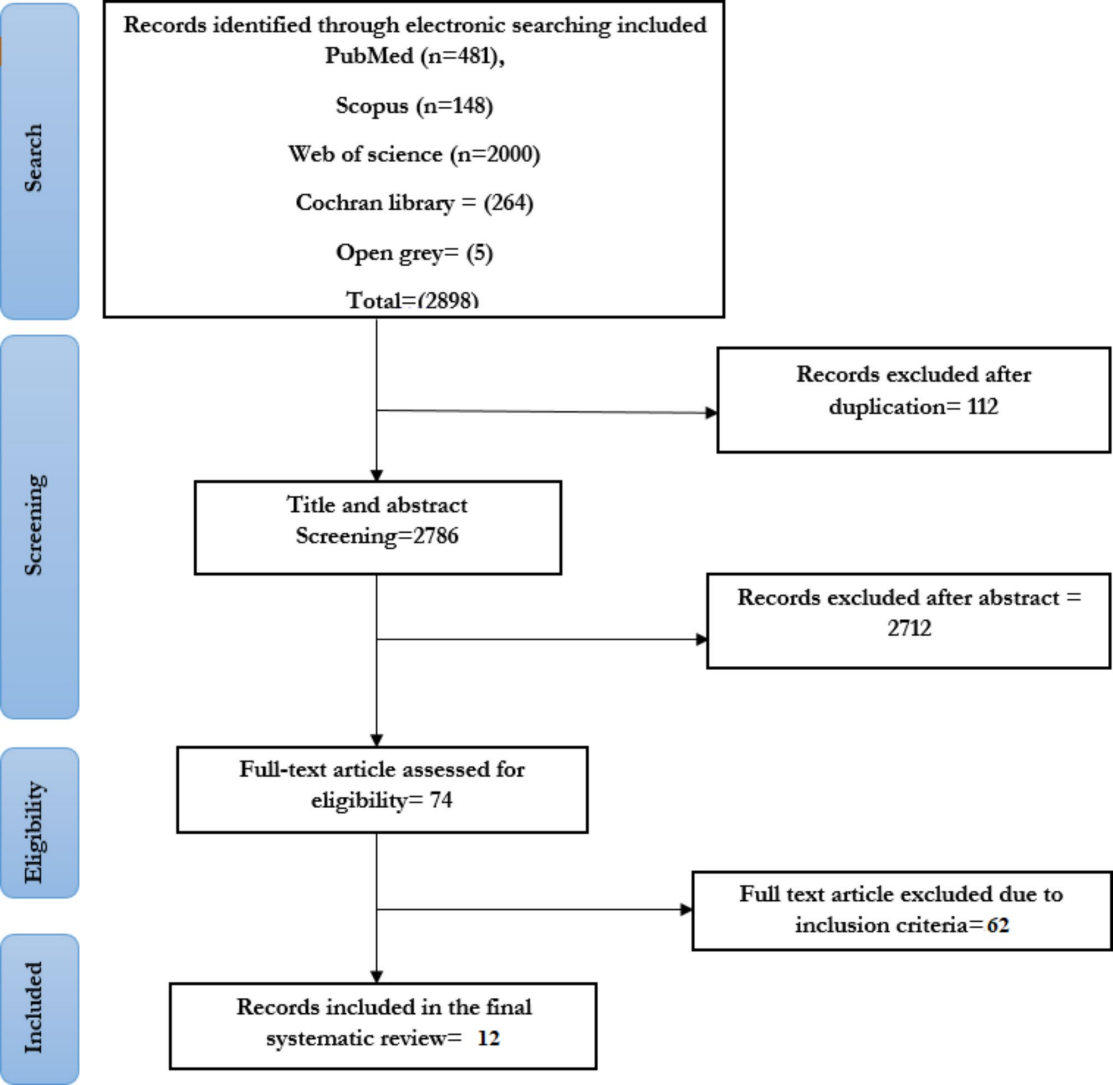
Flowchart demonstrates studies selection process

A total of 2898 studies were retrieved in the initial search; PubMed (n = 481), Scopus (n = 148), ISI web of science (n = 2000), Cochran library (n = 264), and open gray (n = 5). One hundred twelve papers were excluded for duplication. After assessing the title and abstract, 2712 studies were excluded 0.74 papers remained for assessing eligibility criteria. After evaluating studies based on inclusion and exclusion criteria, 62 articles were removed from the search list. In total, 12 RCTs were included this systematic review. Figure 1 demonstrates the study’s selection process.

### Study characteristics

Table 1 shows the basic characteristics of the studies. All included articles were a parallel design published between 2010 and 2020. Most studies were carried out in the United States (n = 5), two in Kenya and Uganda, 1 in Canada, 1 in Brazil, and 1 in Malaysia. Approaches for statistical analysis were intention-to-treat and per-protocol in 1 article, intention-to-treat approach in 7 articles, and not specified in 5 articles. The study sample size varied from 29 to 538, and the total number of patients was 2115. The majority of trials focused on 18 years or older. Eleven studies enrolled both sexes, while one was conducted only on men. The most prolonged follow-up period in the studies was 12 months, and the shortest was one month. Most studies reported a follow-up period of six-month (n = 5) or 12 months (n = 5).

**Table 1:** Characteristics of the included studies

Author	Year	Study design	Country	Sample size(N)		Gender (%)	Mean age, year	Telehealth		Follow up, month	Outcome measurement tool
				Intervention	control	Male		intervention	control		
Sherman EM [38]	2020	Parallel	USA	49	45	61.7%	39.2	One-way daily text message reminders	standard of care with verbal reminders	1,3,6,12	visual analogue scale
Ramsey SE [43]	2021	Parallel	USA	27	26	71.7%	46.7	coaching delivered via the application	coaching delivered via health worker	1, 3, 6, 12	Self‑reported/ HIV viral load
MacCarthy S [58]	2020	Parallel	Uganda	40	59	47.61%	15–24 #	weekly text informing of their adherence level in the previous week	standard care	3	Wise pill device
MacCarthy S [58]	2020	Parallel	Uganda	56	59	47.61%	15–24 #	weekly text information about their own adherence as well as information about the adherence level of their peers in the intervention	standard care	3	Wise pill device
Lester RT [40]	2010	Parallel	Kenya	273	265	34.57%	36.6	SMS intervention	standard care	6,12	self-reported adherence / HIV viral load
Kurth AE [41]	2019	Parallel	Kenya	118	118	21.61%	37.5	CARE + Kenia computer-based counseling program	standard care	3, 6,9	HIV- viral load
Kurth AE [44]	2016	Parallel	USA	253	241	59.91%	47.8	CARE + Spanish computer-based counseling program	standard care	3, 6, 9, 12	HIV- viral load
Glasner S [45]	2020	Parallel	USA	17	18	84%	50.2	computer-based counseling and text messaging	standard care	1, 2, 3	unannounced phone-based pill counts / HIV viral load
Côté J [14]	2020	Parallel	Canada	45	43	82.95%	41.5	web-based intervention	standard care	6	HIV viral load / self-administered questionnaire
Da Costa TM [42]	2012	Parallel	Brazil	14	15	0	34.6	SMS message	standard care	1,2,3, 4	self-reported adherence/Pill counting/ MEMS
Claborn KR [26]	2014	Parallel	USA	43	49	86.95%	42.8	electronic life steps	standard care	1	Self-reported adherence/CD4 + count
Abdulrahman SA [39]	2017	Parallel	Malaysia	121	121	88.84%	32.8	weekly medication reminder SMS and telephone call reminders	standard care	3,6	self-administered Adult AIDS Clinical Trial Group (AACTG) adherence questionnaires

#Mean or Median not reported

### Assessment of risk of bias

As shown in Tables 2 and 8 out of 12 studies were classified as ‘good,’ and four were assessed as ‘fair’. An unclear risk of bias was revealed in some key domains. Eight studies had no information on allocation concealment, which may have led to selection bias. Three studies were subject to performance bias due to the lack of blinding of study participants and personnel. One study was vulnerable to detection bias due to a lack of blinding of assessors or analysts. There was selective outcome reporting in 2 studies.

**Table 2:** _Quality of bias assessment of the included studies according to the Cochrane guidelines

Author name, references	year of publication,	Random sequence generation	Allocation concealment	Blinding of participants and personnel	Blinding of outcome assessment	Incomplete outcome data	Selective reporting	Overall quality
Sherman EM [38]	2020	L	U	U	U	L	L	Good
Ramsey SE [43]	2021	L	U	H	U	L	L	Good
MacCarthy S [58]	2020	L	U	U	U	L	H	Fair
MacCarthy S [58]	2020	L	U	U	U	L	H	Fair
Lester RT [40]	2010	L	L	H	L	L	L	Good
Kurth AE [41]	2019	L	U	U	U	L	L	Good
Kurth AE [44]	2016	L	L	U	U	L	L	Good
Glasner S [45]	2020	U	U	H	H	L	L	Fair
Côté J [14]	2020	L	L	L	L	L	L	Good
Da Costa TM [42]	2012	L	U	L	U	L	L	Good
Claborn KR [26]	2014	U	U	U	U	L	L	Fair
Abdulrahman SA [39]	2017	L	L	U	L	L	L	Good

### Meta-analysis

Among our results, the highest (1.47, CI 0.78 to 2.77) and lowest (0.96, CI 0.64 to 1.46) OR were reported in Sherman EM et al. and Côté J et al. studies, respectively [14, 38]. Likewise, the highest (1.16, CI 0.89 to 1.43) and lowest (-0.03, CI -0.44 to 0.38) SMD were reported by Abdulrahman SA et al. and Claborn KR et al [29, 39].

**Fig. 2:**
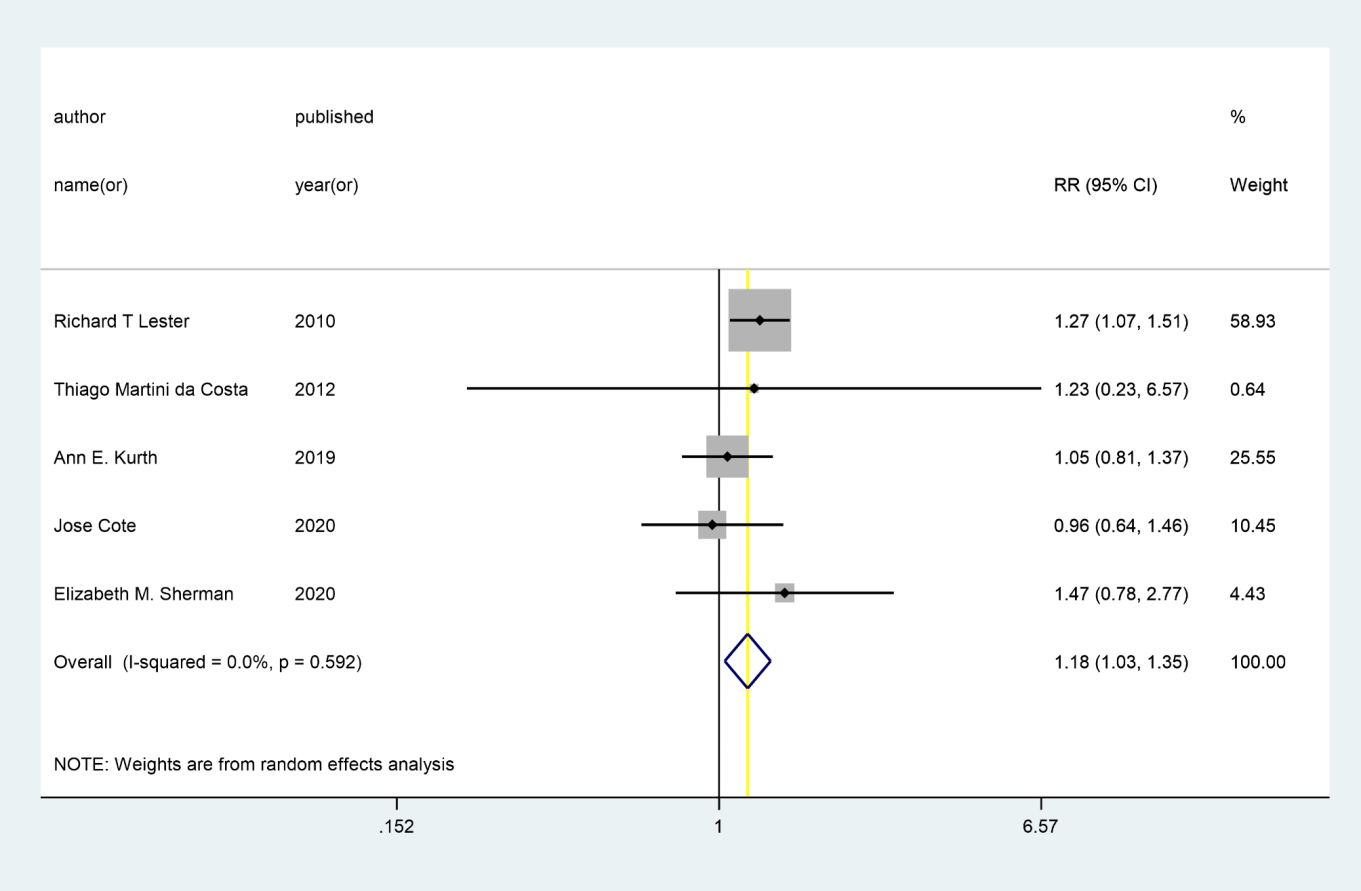
Forest plot showing the pooled Risk Ratio of telehealth effect on ART adherence of treatment in patients with HIV, using random effects model

**Fig. 3:**
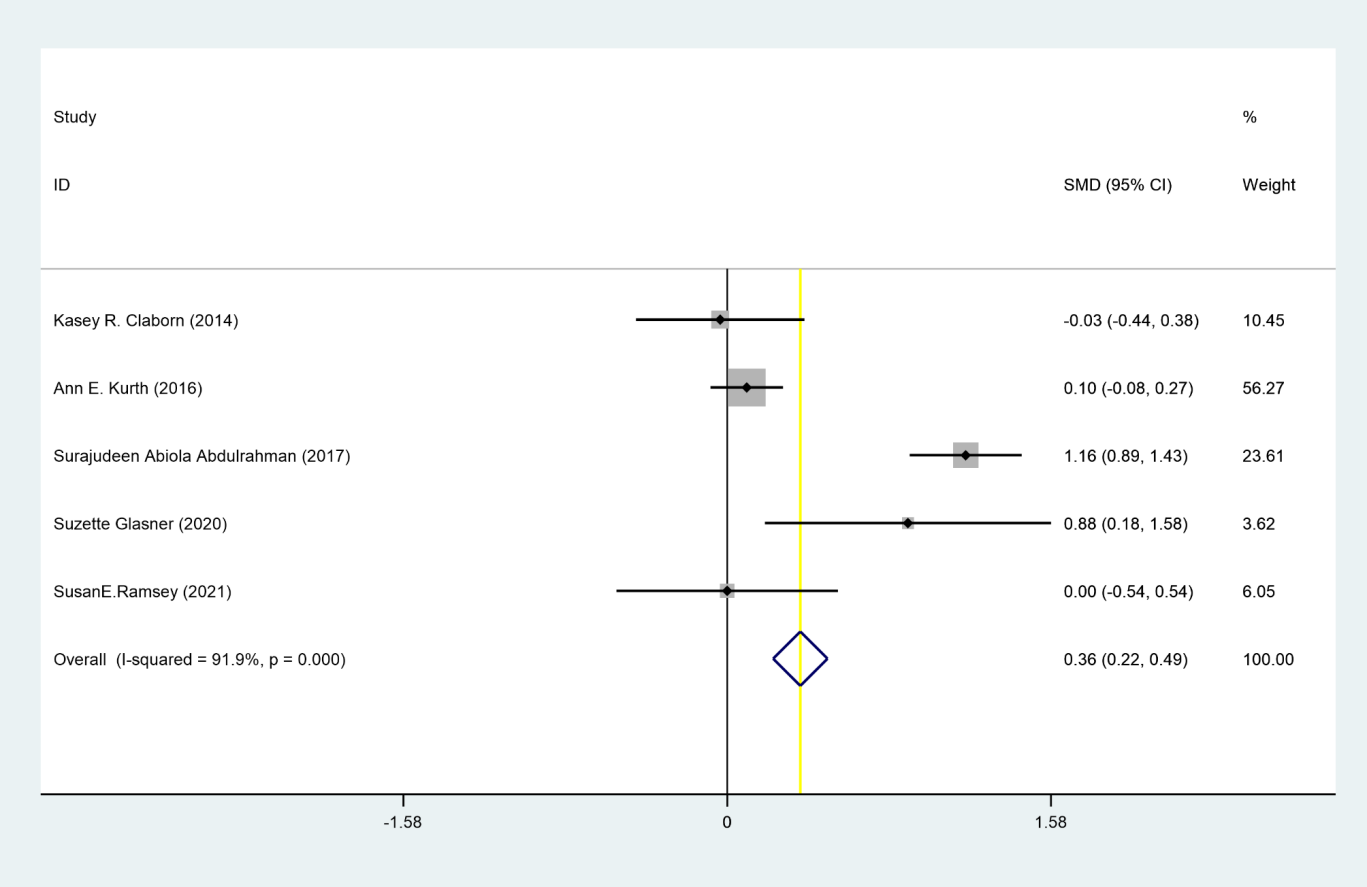
Forest plot showing the pooled standard mean difference of telehealth effect on adherence of treatment in patients with HIV, using random effects model

A random-effects meta-analysis using Five RCTs yielded the pooled RR estimate of 1.18 (95% CI: 1.03 to 1.35, p < 0.05); I^2^ = 0, suggesting the adherence to treatment among patients with HIV who received telehealth intervention was significantly 18% upper than control groups [14, 38, 40] [41, 42]. Moreover, the random effects analysis of SMD showed a positive effect for telehealth with SMR = 0.36 (95% CI: 0.22 to 0.49, p < 0.05); I^2^ = 91.9% indicating that telehealth intervention increased ART adherence to treatment compared to the control group [26, 39, 43–45]. (Figures 2 and 3)

## Discussion

Numerous patients reported isolation, stigma, depression, and other social issues due to HIV diagnosis [46]. Researchers have recommended telehealth to reduce these complications and increase access to treatments and the necessary follow-up for adherence in HIV-positive patients. In the context of the COVID-19 pandemic, due to insufficient information on the treatment of the virus, many governments applied public health policies such as using face masks, social distancing, quarantine, and isolation, which can lead to discontinuous ART [47, 48]. A widespread and prolonged interruption in the supply of ART could increase the HIV mortality rate [49, 50]. Therefore maintaining ART during any disruptions in the health system is the critical priority for HIV programs [51]. According to Jewell BL et al., A three-month interruption in HIV services lead to the same number of exceeding deaths as those that might be saved through social distancing. Also, nine months of ART in 6–90% of individuals could cause more number of death due to HIV than COVID-19 deaths [52].

These conditions contributed to the wide use of telehealth in the screening, diagnosis, and follow-up of health conditions. Several studies have evaluated the effect of telehealth in different treatment and follow-up stages in patients living with HIV [22–24, 53].

To the best of the authors’ knowledge, this study is the first meta-analysis of RCT studies, indicating the pooled estimates of Risk Ratio (RR) and Standard Mean Differences (SMD) measures for evaluating the potential impact of telehealth on ART adherence in patients with HIV.

The present systematic review identified twelve eligible RCT studies, ten of which were included in the meta-analysis. The researchers calculated the mean and standard deviation for five RCTs and the Risk Ratio and CI of 95% for the rest of the studies. Although three studies demonstrated a non-significant positive effect of telehealth, one showed a non-significant negative effect among studies that reported the Risk Ratio. Of the five RCTs that reported SMD, two showed the effectiveness of telehealth in enhancing ART treatment. However, one RCT reported a non-significant reverse relationship between intervention and comparison groups. One-fifth of the studies demonstrated a non-significant difference between the experiment and control groups.

The pooled RR estimate indicated that patients with HIV who received telehealth intervention had an upper ART adherence (1.18 times) than the control group. Likewise, pooled SMD measure was 0.36 (95% CI: 1.03 to 1.35). This study pooled data from 10 RCTs, showing a significant positive role of telehealth in ART adherence of HIV patients. Various studies have demonstrated close results with our findings [54, 55]. According to Shah R et al., there was a significant moderate effect of text message intervention in adherence of HIV patients to ART(SMD = 0.42 (0.03 to 0.81) p = 0.04) [56].

In contrast to our findings, the results of Claborn KR et al., which investigated the effect of an electronic intervention to promote HIV medication adherence, showed a lack of efficacy in medication adherence (p = 0.92). These results revealed a trend in increasing adherence over time among telehealth interventions, while participants in the standard care condition remained steady between baselines and follow-up [26].

Variation of the effect size mainly depends on different age groups, methodology, quality of the study implementation, culture, and the context of the study. Moreover, different telehealth interventions and follow-up periods may affect ART adherence in various studies. Claborn KR et al. and Lester RT et al. reported one and twelve-month Follow-up periods, respectively. Richard Kas Lester et al., unlike Kasey R. Claborn et al., reported that telehealth improves ART adherence among patients with HIV [26, 40]. M Grove et al. reported that effective telehealth reduces stigma-related delays in care and transportation-related financial problems. Likewise, improved mental health and quality of life were reported as the outcomes of good adherence to ART through telehealth [57].

Studies that have been conducted to investigate the effect of telehealth on ART adherence among HIV patients were included in the present study. Regarding the different follow-up periods and the specific method of telehealth used in various studies, high heterogeneity and controversy were reported in the present study. Therefore, it is recommended to carry out more studies on the impact of various telehealth methods on the adherence of HIV patients.

### Limitations

This review is the first meta-analysis demonstrating the impact of telehealth on improving treatment adherence in patients with HIV. However, our study had limitations. First, our systematic electronic search was restricted to evidence published in English. However, the abstract of the articles in other languages is often published in English. We found no study abstract in other languages that could be met the present study inclusion criteria. Second, given that most HIV patients live in the WHO African region, studies may have been published in local databases of this region and have been missed in our study. It could lead to a slightly overestimating of present study results.

## Conclusion

Meta-analysis using a random-effects model and pooled estimates of RR and SMD confirmed that telehealth improved treatment adherence in patients with HIV. There is a direct relationship between appropriate treatment adherence and quality of life in patients living with HIV. Telehealth can be used as an alternative to reducing stigma, isolation, and the financial burden of transportation for face-to-face follow-up and visits to manage HIV.

On a large scale, telehealth can be utilized as a supplementary component for ART delivery and retention toward successful adherence to the therapy.

## Data Availability

The datasets generated and analyzed during the current study are available from the corresponding author upon reasonable request.

## Abbreviations

AIDS: Acquired Immune Deficiency Syndrome
ART: Antiretroviral therapy
CIs: Confidence intervals
HIV: Human immune virus
OR: Odds Ratio
RCTs: Randomized Controlled Trials
RR: Risk ratio
SMD: Standard Mean Differences
COVID-19: Coronavirus Disease 2019
PRISMA: Preferred Reporting Items for Systematic Reviews and Meta- Analysis
